# Optimizing T Cell-Based Therapy for Glioblastoma

**DOI:** 10.3389/fimmu.2021.705580

**Published:** 2021-08-05

**Authors:** Aida Karachi, Farhad Dastmalchi, Saina Nazarian, Jianping Huang, Elias J. Sayour, Linchun Jin, Changlin Yang, Duane A. Mitchell, Maryam Rahman

**Affiliations:** Lillian S. Wells Department of Neurosurgery, University of Florida (UF) Brain Tumor Immunotherapy Program, University of Florida, Gainesville, FL, United States

**Keywords:** T cell dysfunction, exhaustion, glioblastoma, glioma, CAR T cells, adoptive T cell transfer

## Abstract

Evading T cell surveillance is a hallmark of cancer. Patients with solid tissue malignancy, such as glioblastoma (GBM), have multiple forms of immune dysfunction, including defective T cell function. T cell dysfunction is exacerbated by standard treatment strategies such as steroids, chemotherapy, and radiation. Reinvigoration of T cell responses can be achieved by utilizing adoptively transferred T cells, including CAR T cells. However, these cells are at risk for depletion and dysfunction as well. This review will discuss adoptive T cell transfer strategies and methods to avoid T cell dysfunction for the treatment of brain cancer.

## Introduction

T cells are the key players of the adaptive immune response, and their potency is being leveraged for the treatment of cancer. T cells represent a diverse population of immune cells in the peripheral blood and lymphoid organs, and their overarching function is to rid the host of “non-self” or antigen expressing cells. Many studies have shown a positive correlation between the presence of tumor infiltrating T cells and prognosis in solid tissue malignancies (1–3) including glioblastoma (GBM) (4). T cell immunotherapeutic platforms have had success in certain hematologic and solid tissue cancers (5). However, T cell dysfunction is a major limitation for the efficacy of these strategies in the treatment of GBM (6).

**Figure 1:**
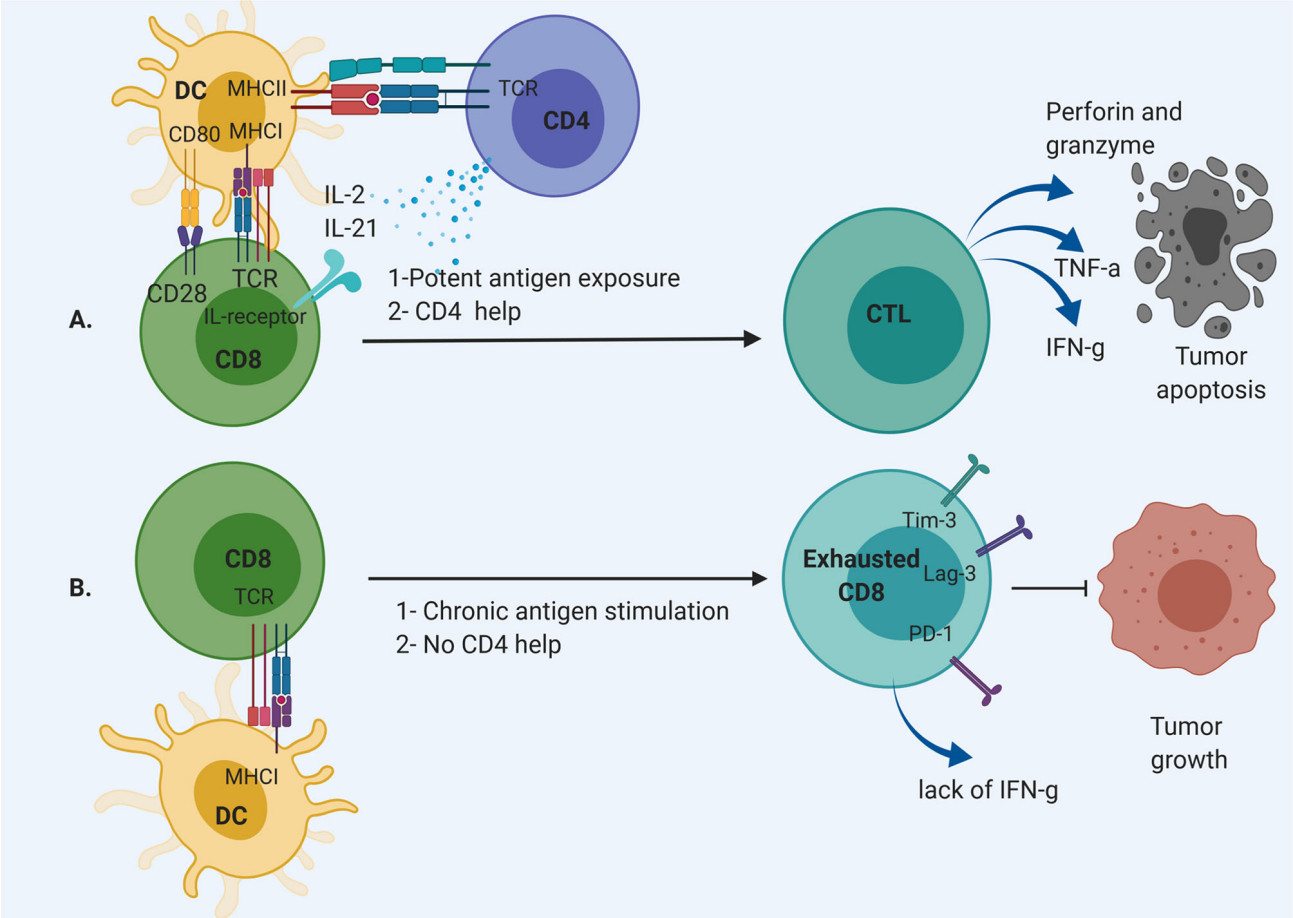
CD8 T cell differentiation pathway. **(A)** CD8 T cell are activated through MHC I by dendritic cells (DCs) with CD4 “help” *via* IL-2 and IL-21 secretion. This results in the development of CD8 effector T cells that can cause cytolysis. **(B)** Chronic antigen exposure and lack of appropriate support from T helpers result in exhausted CD8 T cells without effector function to remove tumor cells.

Dysfunctional T cells are defined by loss of effector function, including loss of cytotoxicity, decreased secretion of inflammatory cytokines such as interleukin-2 (IL-2), tumor necrosis factor-*α* (TNF-*α*), or interferon- γ (IFN-γ) (7). These cells often develop due to chronic antigen exposure with loss of the ability to respond to antigen with cytolysis **(** **Figure 1** **).** Dysfunctional T cells can limit the efficacy of immunotherapeutic strategies for patients with GBM. Infusion of potent T cells educated against particular antigens is an attractive strategy to overcome the dysfunction and sequestration seen in host T cells in patients with GBM. Several platforms are being developed, including adoptive transfer of autologous T cells followed by vaccination and autologous T cells engineered for improved anti-tumor efficacy such as chimeric antigen receptor (CAR) T cells. These platforms have shown some response rates in early trials. However, these therapies are limited by issues with engraftment, a hostile tumor microenvironment, and induced T cell dysfunction. In this review, we will discuss adoptive transfer of T cells for the treatment of GBM and factors that affect the potency of these approaches.

## Exogenous T Cells for GBM

**Figure 2:**
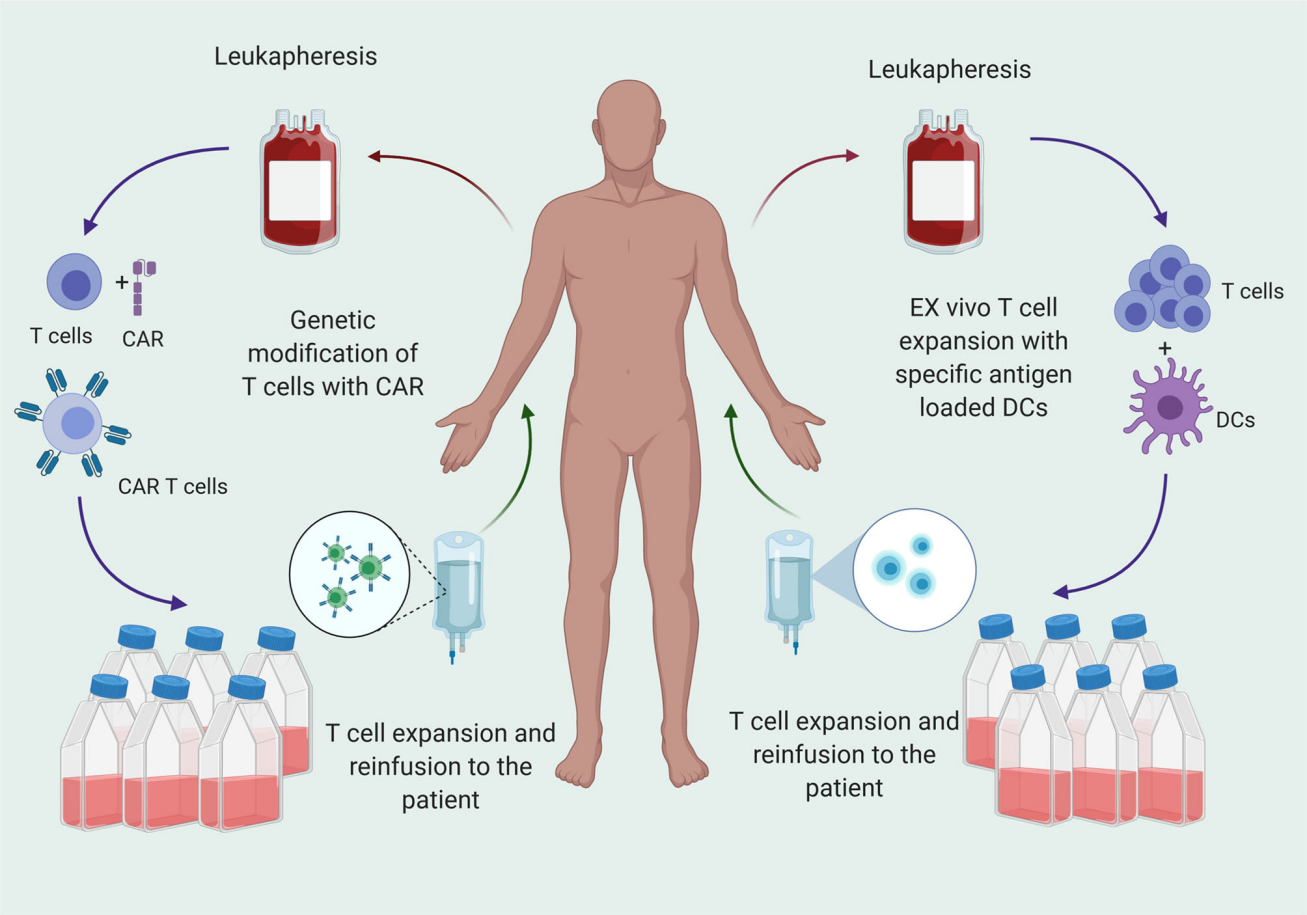
T cell therapy for cancer treatment is transfer of T cells that are specific for tumor antigens to the patients after *ex vivo* expansion. T cells are matured from peripheral blood mononuclear cells (PBMCs) and primed against antigen or genetically engineered to express CARs that specific for the antigen and reinfused to patients after *ex vivo* expansion.

Adoptive T cell infusion provides the host with a bolus of functional T cells primed against a particular antigen. Approaches to generate tumor specific T cells are 1) infusion of autologous, expanded T cells primed against the antigen of interest, or 2) infusion of autologous, engineered T cells such as chimeric antigen receptor (CAR) T cells **(** **Figure 2** **).** Autologous T cells are typically harvested from the peripheral blood (8). Tumor infiltrating lymphocytes (TILs) are intrinsically tumor specific, but in patients with GBM, are too few and dysfunctional, and therefore do not represent a viable source of cells (9, 10). Peripheral circulating T cells must be primed against antigens by co-culturing with antigen loaded dendritic cells (DCs) or through genetic engineering. After detecting antigen specific T cell clones, these cells are expanded and infused into the patients as adoptive T cell transfer.

These strategies are being tested in early phase trials (**Table 1**). A phase I/II study (NCT00331526) in 2004 studied the safety of implantation of lymphocytes into the tumor resection cavity in patients with newly diagnosed or recurrent glioma (11, 12). The lymphocytes were generated from PBMCs and grown with IL-2. The investigators called these cells lymphokine activated killer (LAK) cells. They found that this approach was safe in 40 patients. However, the analysis did not evaluate the engraftment or persistence of the cells. An on-going phase I/II study is testing autologous cytotoxic T cells primed against pp65 CMV antigen in patients with newly diagnosed GBM (NCT02661282). In this study, patients receive dose intensified temozolomide to induce lymphopenia and leverage the homeostatic lymphoproliferation that ensues after temozolomide induced lymphopenia. Patients receive up to 4 cycles of temozolomide followed by T cell infusion. The results so far have found that the production of large numbers of T cells from patients with GBM is feasible, and the treatment has been safe. Results on engraftment and clinical efficacy are still underway (13). The ERaDICATe clinical trial (NCT00693095) is also testing CMV targeting T cells with temozolomide and is adding DC vaccines to one of the cohorts to determine if this improves the persistence of T cells (14).

**Table 1:** T cell clinical trials for brain tumors.

Title/ Trial NCT	Phase	Disease	T cell product	Interventions	n	OS	Status	Reference
Cellular Adoptive Immunotherapy in Treating Patients with Glioblastoma Multiforme (NCT00331526)	II	Brain and CNS tumors, newly diagnosed or recurrent glioma	PBMC derived lymphocytes grown with IL-2 (lymphokine activated killer cells)	**Biologic:** 1. Aldesleukin	86	Median survival of 20.5 months with a 1-year survival rate of 75%)	Completed	(11) (12)
Autologous CMV-Specific Cytotoxic T Cells and Temozolomide in Treating Patients with Glioblastoma (NCT02661282)	I/II	Newly diagnosed CMV positive GBM	*Ex vivo* expanded polyclonal CD8+ and CD4+ CMV T cells from peripheral blood of GBM patients	**Drug:** 1. Dose-intensified Temozolomide	65 (34 were screened)	N/A	Active, not recruiting	(13)
Evaluation of Recovery from Drug-Induced Lymphopenia Using Cytomegalovirus-specific T-cell Adoptive Transfer (ERaDICATe) (NCT00693095)	I	GBM	CMV-autologous lymphocyte transfer	**Biologic:** 1. CMV-DC vaccine	22	N/A	Completed	(14)
Adoptive Cellular Therapy in Pediatric Patients with High-grade Gliomas (ACTION) (NCT03334305)	I	GBM	Total tumor RNA primed autologous T cells	**Biologic:** 1. TTRNA-DC vaccines with GM-CSF 2.Autologous Hematopoietic Stem cells (HSCs) **Drug:** 1. Dose-intensified Temozolomide 2.Td vaccine	18	N/A	Recruiting	NCT03334305
Brain Stem Gliomas Treated With Adoptive Cellular Therapy During Focal Radiotherapy Recovery Alone or With Dose-intensified Temozolomide (BRAVO) (NCT03396575)	I	Diffuse intrinsic pontine glioma (DIPG)	Total tumor RNA primed autologous T cells	**Biologic:** 1. TTRNA-DC vaccines with GM-CSF 2.Autologous hematopoietic stem cells (HSCs) **Drug:** 1. Cyclophosphamide + Fludarabine 2.Td vaccine	21	N/A	Recruiting	NCT03396575
CAR T Cell Receptor Immunotherapy Targeting EGFRvIII for Patients with Malignant Gliomas Expressing EGFRvIII (NCT01454596)	I/II	Recurrent GBM	A single infusion of EGFRvIII CAR T cells	**Drug:** 1. Aldesleukin 2. Fludarabine 3. Cyclophosphamide	10	8	Completed	(20)
Genetically Modified T-cells in Treating Patients with Recurrent or Refractory Malignant Glioma (NCT02208362)	I	Recurrent or Refractory GBM	Intratumoral Infusion of IL13R alpha 2-specific CAR T cells followed by infusions into the ventricular system	**N/A**	92	N/A	Recruiting	(21)
IL13Ralpha2-Targeted Chimeric Antigen Receptor (CAR) T Cells with or Without Nivolumab and Ipilimumab in Treating Patients with Recurrent or Refractory Glioblastoma (NCT 04003649)	I	Recurrent or Refractory GBM	Intratumoral Infusion of IL13R alpha 2-specific CAR T cells followed by infusions into the ventricular system	**Drug:** 1. Ipilimumab 2. Nivolumab	60	N/A	Recruiting	NCT 04003649
CMV-specific Cytotoxic T Lymphocytes Expressing CAR Targeting HER2 in Patients With GBM (NCT01109095)	I	Recurrent GBM	Infusion of autologous CMV-specific cytotoxic T-lymphocytes genetically modified to express CAR19 targeting the HER2 molecule	**N/A**	17	24.5	Completed	22
3rd Generation GD-2 Chimeric Antigen Receptor and iCaspase Suicide Safety Switch **(GRAIN)** (NCT01822652)	I	Relapsed or refractory Neuroblastoma	Infusion of third generation GD2-CAR (GD2-CAR3) generated from patients’ PBMC	**Drug:** 1. Cyclophosphamide 2. Fludarabine 3. Pembrolizumab	11	16.8	Active, not recruiting	(41)
Pembrolizumab in Patients Failing to Respond to or Relapsing After CAR T Cell Therapy for Relapsed or Refractory Lymphomas (NCT02650999)	I/II	CD19 Diffuse Large B-cell Lymphomas, Follicular Lymphomas, Mantle Cell Lymphomas	Infusion of PBMC derived CAR T cells specific for CD19	**Drug:** Pembrolizumab	12	N/A	Active, not recruiting	(42)
Study of DC Vaccination Against Glioblastoma (NCT01567202)	II	GBM	Infusion of DC vaccine loaded with glioblastoma stem cell-like (GSC) antigens	**Biologic:** 1. DC vaccination **Drug:** 1. Temozolomide **Radiation:** 1. Radiotherapy	43	13.7	Recruiting	
Chemotherapy, Radiation Therapy, and Vaccine Therapy With Basiliximab in Treating Patients With Glioblastoma Multiforme That Has Been Removed by Surgery (NCT00626015)	I	GBM	N/A	**Biologic:** 1.PEP-3-KLH conjugate vaccine **Drug:** 1. Daclizumab 2. Temozolomide	16		Completed	NCT00626015
EGFRvIII CAR T Cells for Newly-Diagnosed WHO Grade IV Malignant Glioma (NCT02664363)	I	GBM	EGFRvIII CAR T cells	**Drug:** 1. Dose-intensified temozolomide	3	N/A	Terminated	NCT02664363

CMV, cytomegalovirus; DC, dendritic cells; TTRNA, Total tumor RNA; GM-CSF, Granulocyte-macrophage colony-stimulating factor; TD vaccine, tetanus; diphtheria vaccine.

Similarly, adoptive transfer of clonally selected T cells targeting tumor has been described for the treatment of recurrent primitive neuroectodermal tumors (PNETs) (19). Blood was drawn and tumor biopsies were obtained from 10 patients for vaccine preparation. Autologous T cells were isolated from patients’ blood and were primed and expanded *ex vivo* by exposure to dendritic cells loaded with total tumor RNA extracted from tumor biopsies. The T cells were infused back to the patients after conditioning chemotherapy. Some patients received non myeloablative chemotherapy, and others received myeloablation followed by stem cell rescue. These conditioning regimens have significant implications for the potential for T cell proliferation and are further discussed in the following sections of this review. Patients subsequently received three dendritic cell (DC) vaccines. T cell receptor (TCR) RNA sequencing of peripheral blood mononuclear cells (PBMCs) after treatment demonstrated a large clonal expansion of T cells, which correlated with clinical outcomes. This platform is now also being tested in high-grade pediatric glioma (NCT03334305) and diffuse intrinsic pontine glioma (DIPG) (NCT03396575).

Alternatively, T cells can be engineered to provide a more potent population of cells. Autologous patient derived T cells isolated from patients can be modified with a CAR gene. CAR T cells are designed to express artificial T cell receptor using viral transfection to recognize cancer antigens. CARs are composed of extracellular, transmembrane and intracellular domain. The extracellular domain, also known as tumor targeting domain, is composed of single chain variable fragment (scVF) that is made up of the variable regions of the heavy and light chains (20). The tumor targeting domains are not restricted by MHC bound antigens. They can recognize non-MHC cell surface proteins. Intracellular domain composed of CD3ζ to direct T cells for performing the primary cytolytic activity. However, cytotoxic T cells require further signaling when they encounter a cognate foreign antigen to induce expansion, persistence, and cytokine secretion (21). To address this issue, 2^nd^ and 3^rd^ generation of CAR T cells developed with the 2^nd^ generation composed of co-stimulatory domains such as CD28, 4-1BB to improve proliferation and cytokine production of CAR T cells. The 3^rd^ generation of CAR T cells, composed of multiple signaling domains such as CD28 and 4-1BB or Ox40. The most current generation CAR T cells, T cells redirected for universal cytokine-mediated killing (TRUCKS), have co-stimulatory molecules and are armed with transgenes to express a synthetic protein of interest such as immune stimulatory cytokines of IL-2, IL-5, IL-12 to exhibit an improved anti-tumor function and resistance to immunosuppressive tumor microenvironment (22, 23).

Two FDA approved CAR T cell therapies are available for treatment of acute lymphoblastic leukemia (ALL) and diffuse large B cell lymphoma (DLBCL), Tisagenlecleucel (CTL019, Kymriah^©^) and axicabtagene ciloleucel (Yescarta^©^). Three single antigen CAR T cell therapies are under investigation for GBM targeting EGFRvIII, IL13Rα2, and HER2. A single infusion of EGFRvIII CAR T cells was tested in 10 patients with recurrent GBM in a phase I study (NCT01454596) that required EGFRvIII expression in tumor samples (15). Patients did not receive conditioning with chemotherapy prior to infusion. Cells were detectable by flow cytometry or PCR but declined significantly (2-10 fold) within 14 days post infusion. The level of existing lymphopenia did not correlate with peak engraftment. EGFRvIII CAR T cells were detectable in the tumor specimens of patients who had early surgery after infusion (within two weeks). However, some of the specimens also had infiltration of immunosuppressive regulatory T cells and upregulation of IDO1, PD-L1, and IL-10, suggesting that the infiltration of EGFRvIII cells within the tumor incited a compensatory immunosuppressive response.

IL13Rα2 targeting CAR T cells are also being tested in patients with recurrent GBM either as monotherapy (NCT02208362) or in combination with immune checkpoint blockade (NCT 04003649). A report of a single patient with multiple intracranial and spinal lesions of recurrent, wide-spread GBM demonstrated regression when treated with intrathecal delivery of IL13Rα2 CAR T cells developed from autologous cells (16). The patient eventually succumbed to disease progression. Similarly, HER2 CAR T cells were tested in a phase I study in patients with recurrent GBM with HER2 expression (24). No conditioning regimen was given prior to infusion. Most patients had the highest concentrations of detectable HER2 CAR T cells in the peripheral blood within two weeks of infusion. Six weeks after infusion, detectable CAR T cells declined significantly. The median overall survival was 24.5 months after diagnosis and was 11.1 months after T cell infusion.

## Barriers to T Cell-Based Therapy

### Engraftment

A major hurdle for T cell therapy is the engraftment of cells. Engraftment for T cell therapy in brain tumors refers to presence of the cells in the peripheral blood and migration within the tumor microenvironment for sustained anti-tumor responses. This definition is different from the traditional concept of engraftment of hematopoietic stem cells, which are expected to take residence in the bone marrow for cellular production. The kinetics of autologous T cells after the infusion is a decline as they distribute in the tissues, an increase as they proliferate, and a subsequent decline that persists (25, 26). As discussed in the previous section, most patients do not have detectable levels of infused T cells within two weeks after infusion. Approaches to improve engraftment are pre-conditioning with chemotherapy to induce lymphopenia. This allows for the infused T cells to have less “competition” for cytokines and also to leverage the homeostatic lymphoproliferation that results from the lymphopenia (27–29). Another alternative strategy is the use of DC vaccination following T cell infusion. In a pilot study, 17 patients with newly diagnosed GBM were randomized to receive CMV targeting T cells alone or with DC vaccines (14). The patients who received vaccines had significant increases (~1.5-fold, p=0.04) in T cells that expressed IFN gamma, TNF alpha, and CCL3. However, this analysis was performed only seven days after T cell infusion. Therefore, the persistence of cells after DC vaccination was not evaluated.

Another method to overcome issues with loss of T cell frequency in the circulation is to force cells to accumulate in the tumor microenvironment. This technique was utilized successfully in pre-clinical models testing a CD70 CAR T cell (30). The CD70 CAR T cell was modified to express IL-8 receptors (CXCR-1 and CXCR-2) (31). In the setting of CD70 expressing tumors treated with radiotherapy (RT), the modified CD70 CAR T cells had much greater trafficking to the tumors due to IL-8 upregulation after RT. The improved CAR T cell tumor infiltration resulted in long term survivors compared to only 35 days of survival in untreated animals, and a strong memory T cell response that prevented regrowth of tumors on re-challenge.

### Potency

One of the limiting factor of transferred T cells’ potency is exhaustion. Exhaustion is a T cell state that develops gradually in both transferred and host T cells due to repeated stimulation of the T cell from persistent antigen exposure (32). Exhaustion has distinct signatures but one of the most important characteristics of exhausted T cells is persistent over-expression of inhibitory checkpoints. The over-expression of immune checkpoints is also present in activated T cells. However, activated T cells experience a transient upregulation of immune checkpoints while exhausted T cells have a persistent upregulation of immune checkpoints.

Exhausted T cells are heterogeneous and include two different cell populations: progenitor exhausted T cells and terminally differentiated exhausted T cells. Progenitor exhausted T cells can be generated from both effector T cells or directly from naïve T cells. Progenitor exhausted T cells have some stem cell like characters similar to central memory T cells as they have the potential to proliferate and expand and also reverse to effector T cells after vaccination or PD-1 blockade (33, 34). Although these cells have a high expression of PD-1 and T cell factor-1(TCF-1), which is a self-renewal marker, they have a limited expression of other inhibitory molecules and lack expression of markers like Tim-3 (35).

Terminally differentiated exhausted T cells are generated from high PD-1 expressing cells with expression of multiple immune checkpoints and lack of responsiveness to immune checkpoint blockade (36). Persistent antigen exposure leads to upregulation of transcription factor TOX and alterations of nuclear factor activated T cells (NFAT), which is required for formation of exhausted T cells (37, 38). In GBM, TILs expressing high levels of Tim-3, Lag-3 and PD-1 that fail to secrete IFN-g, IL-2 and TNF-*α* are considered terminally exhausted (9).

Exhaustion can also be seen in transferred CAR T cells that results in reduced anti-tumor efficacy. In elegant experiments performed by Dr. Rao’s group, CD19 reactive CAR T cells were found to have gene expression and chromatin accessibility associated with NFAT pathway including activation of NR4A1-3 (37). When the three NR4A binding motifs were knocked out in the CAR T cells, the gene expression profiles and chromatin regions of effector CD8 T cells were characterized, and they caused tumor regression and prolonged survival in tumor bearing mice (75% in triple knockout CAR T cells versus <5% wild type CAR T cells, p<0.0001).

Other potential strategies to avoid exhaustion of CAR T cells include combining with immune checkpoint blockade (39). Pre-clinical models have demonstrated enhanced anti-tumor efficacy when PD-1 blockade is added to CAR T cells in murine models of lung cancer and breast cancer (40–42). In a small study of patients with neuroblastoma, the addition of PD-1 blockade to lymphodepletive chemotherapy did not enhance the expansion or persistence of third generation GD2 CAR T cells (17). Administration of PD-1 blockade in 10 adult patients with high grade gliomas resulted in blockade of PD-1 on both host T cells and intracranial injected CAR T cells with reduction of PD-1 on T cell surface from 39.3% to 3.8% (18). In this study the effect of PD-1 blockade on T cell function and phenotype were not evaluated. Therefore, T cells engineered to secrete immune checkpoint antibodies are being developed (43, 44).

Another strategy is having the CAR induced only when the antigen is present. Choe et al. developed a CAR T cell that has CAR activation only when a synNotch receptor interacts with the tumor antigen (45). They utilized EGRvIII and myelin oligodendrocyte glycoprotein (MOG) targeting CARs to demonstrate that these particular CAR T cells were more likely to be in a naïve/stem cell memory state. This correlated with better anti-tumor efficacy. NCG mice implanted with GBM6 PDX GBM were treated with α-EGFRvIII synNotch–α-EphA2/IL13Rα2 CAR T cells which resulted in long-term remission of all tumors. In other studies, CAR T cells targeting alkaline phosphatase placental-like 2 (ALPPL2) in murine models of human ovarian and mesotheliomas tumors had longer persistence and better tumor control when synNotch was added (46). Animals bearing M28 mesotheliomas tumors were treated with ALPPL2-synNotch-MCAM CAR T cells and demonstrated complete responses in the majority of animals with less PD-1+/CD39+ exhausted CD8 T cells (~60%) compared to MCAM CAR T cells (~75%).

T cell-based therapies have been limited thus far due to the inability to target all antigen-expressing tumor cells. Strategies to overcome issues with T cell effector function began with the development of 2^nd^ and 3^rd^ generation CAR T cells. First generation CAR T cells only had CD3ζ intracellular domain signaling, which limited the ability of complete activation signaling and secretion of cytokines long-term as the signaling diminished over time (47). Second and third generation CAR T cells added 4-1BB and CD28, which are co-stimulatory factors that improved activation and expansion (22). Fourth generation CAR T cells provide the ability to secrete a protein of interest such as cytokines and chemokines with enhanced T cell persistence and anti-tumor function (23).

In GBM, CAR T cells have also been modified to improve activation. IL13Rα2-CAR T cells were engineered to overexpress IL-15 to enhance effector function (48). Transgenic IL-15 expressing IL13Rα2-CAR T cells had greater expansion and enhanced anti-tumor effector function as measured by cytokine production. IL-15 secreting CAR T cells showed enhanced intracranial persistence with resultant tumor regression in the U373 human glioblastoma orthotopic xenograft mouse GBM model. However, tumors recurred after 40 days due to IL13Rα2 antigen loss (48).

### Antigen Loss

**Figure 3:**
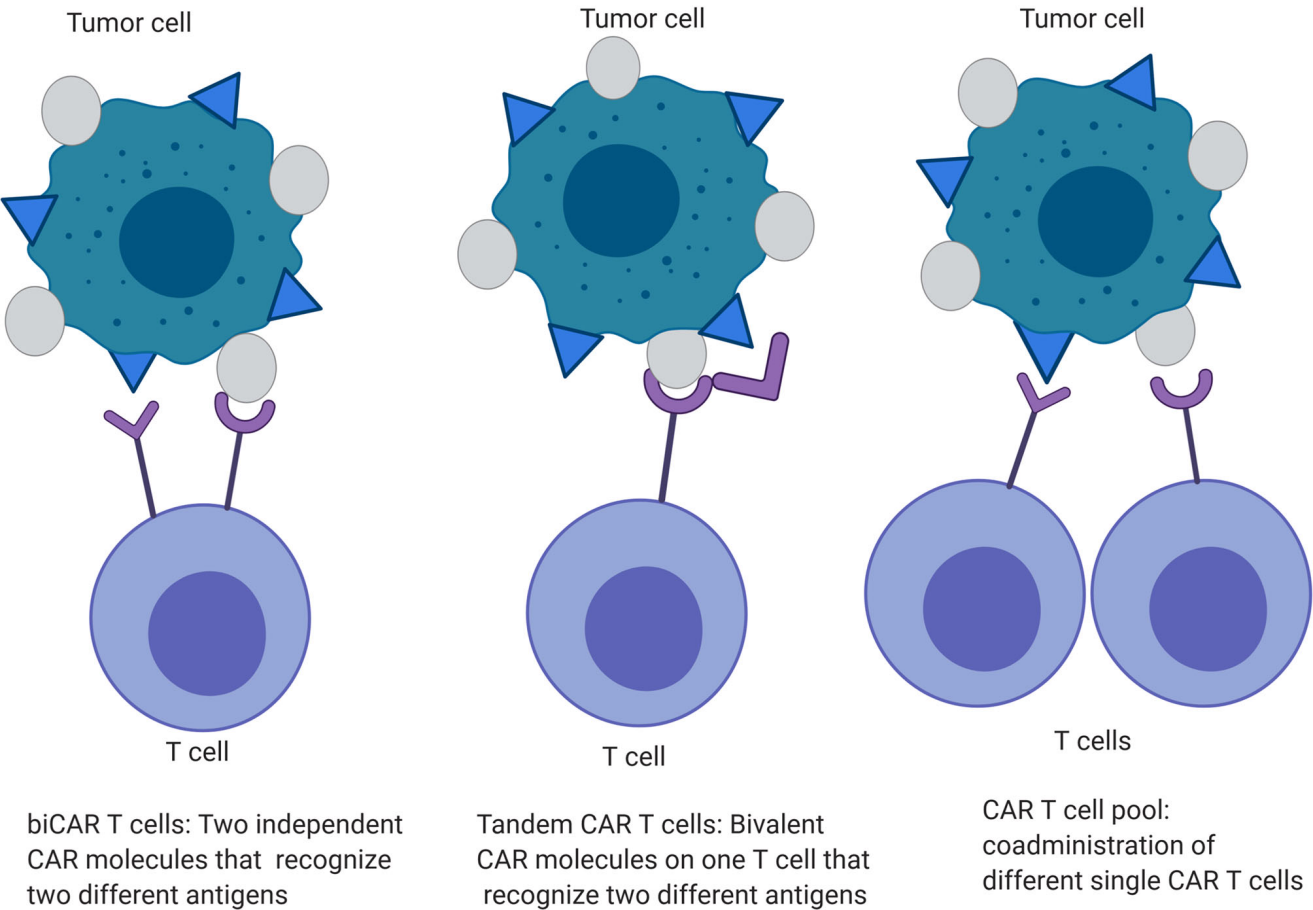
Engineered CAR T cells specific for multiple tumor antigens.

Tandem and trivalent CAR T cells have been developed in an attempt to overcome the issue of antigen loss post CAR T cell therapies that has been seen with both EGFRvIII CAR T cell therapy in patients with GBM (15) and IL13Rα2 CAR T cell therapy in a xenograft GBM mouse model (48). In a murine GBM model, tandem CAR T cells targeting HER2 and IL13Rα (two specific antigen targeting domains within one CAR construct) displayed enhanced activation and anti-tumor function without being more exhaustible than co-expressed HER2 and IL13Rα CAR T cells (biCAR T cells) or pool of single antigen HER2 or IL13Rα CAR T cells (49) (**Figure 3**). These tandem CAR T cells had moderate increases in IFN-gamma and IL-2 secretion and improved tumor-killing capacity (~60% in tandem CAR *vs*~20% in biCAR in U373 model, p<0.05). The animals treated with tandem CAR T cells had a survival of >140 days compared to biCAR (85 days) (p<0.0001).

Due to the heterogeneity of GBM tumor cells, the expression of surface antigens significantly varies between patients, and targeting two antigens may not be an effective treatment for all patients. CAR T cells targeting HER2, IL13Rα2, and ephrin-A2 (EphA2) have been developed to provide antigenic “coverage” for almost all patients (50). This trivalent CAR T cell demonstrated improved anti-tumor activity and survival in GBM patient derived xenografts compared to biCAR (IL13Rα2, andEphA2) and single IL13Rα2 CAR T cells, while lower T cell doses were required to control tumor growth. The ability of the trivalent CAR to overcome tumor antigen loss is still unknown.

Peptide based CAR T cells exploit the binding potential of the peptides to target diverse heterogeneous tumor cells without a shared specific antigen. Researchers complexed a peptide [chlorotoxin (CLTX) extracted from the scorpion’s venom] to the CD28 end of the CAR (51). Although the specific tumor cell surface receptor for CLTX has not been identified, this study found that the CLTX CAR T cells tumor recognition was mediated by expression of membrane-bound matrix metalloproteinase-2 (MMP-2) on the tumor cells. These peptide targeting CAR T cells resulted in anti-tumor effects in orthotopic xenograft models including tumors that did not express typical GBM associated tumor antigens such as IL13Rα2. Therefore, CARs targeting peptides have the potential to overcome the limitations of CARs that target 1 or 2 antigens with recurrence of tumors due to antigen loss.

### Immunosuppressive Microenvironment

In addition to intrinsic problems with infused T cell function, these cells are limited by the immunosuppressive tumor microenvironment (TME). Macrophages and microglia within the murine GBM tumor microenvironment produce CCL2 cytokine to recruit CCR4+ Tregs and CCR2+ Ly6C+ myeloid cells (52). Overexpression of immunosuppressive cytokines and the recruitment of Tregs and myeloid derived suppressor cells (MDSCs) create a hostile environment for engraftment or effector function of cytotoxic T cells (53, 54). This environment is hostile not only locally but also peripherally as T cells have been shown to be sequestered within the bone marrow of patients with GBM (53). These data suggest that T cell egress and trafficking into intracranial malignancies are additional inhibitory mechanisms that must be overcome to initiate and perpetuate a cycle of self-sustaining cancer immunotherapy.

Myeloid cells compose 30-50% of GBM tumor mass and accumulate in the peripheral blood sabotaging the efficacy of T cells (54, 55). Tumor associated myeloid cells express high levels of PD-L1 (55). The majority of PD-L1 expression in the tumor microenvironment results from myeloid cells and not tumor cells (56). In a murine study, radiation therapy was used to upregulate the expression of PD-L1 on myeloid cells to produce synergy when combined with PD-L1 blockade (55). Targeting of myeloid cells and PD-1 expression on T cells leads to reversal of immune resistance to DC vaccination and abundance of T cell infiltration within the tumor with resultant long-term survival in GL-261 GBM bearing mice (56). However, these strategies have not yet been tested in combination with T cell infusion therapies.

Other signaling pathways except than PD-1/PD-L1 are also involved in the dysfunction of T cells mediated by tumor associated myeloid cells. B7 superfamily membrane 1 (B7S1), also known as B7-H4, is an inhibitory molecule expressed by tumor associated myeloid cells that negatively regulates activation of T cells and promotes exhaustion of tumor infiltrating CD8 T cells (57). Inhibition of B7S1 on tumor infiltrating myeloid cells and PD-1 on T cells improves CD8 T cell anti-tumor immune responses in murine cancer models (57). In phase II randomized trial investigating DCs loaded with lysates from GBM cells cultured in stem cell media, patients with low B7-H4 expression had prolonged survival (58). This increase in survival was associated with higher T cell infiltration in the tumors.

Adoptive transfer of autologous T cells is also limited by immunosuppression from circulating Tregs (59–61). Patients with GBM have natural, or thymus derived Tregs and induced Tregs (62, 63). Tregs are associated with reduced survival and are linked to tumor recurrence in patients with GBM (64). IDO expression on tumor cells and CCL-2 secretion from microglia and macrophages in the tumor microenvironment contribute to recruiting Tregs (CD4+CD25+ FOXP3+) (52, 60, 65). Targeting Tregs may have the potential to be synergistic with T cell infusions, but this has not been tested. Treatment targeting Tregs has thus far only been tested alone or in combination with standard chemotherapy or radiation. For example, glucocorticoid-induced TNFR-related protein (GITR) are receptors expressed on Tregs, and antibody blockade of GITR has been shown to have efficacy in murine models (66). Intratumoral treatment with an antibody against GITR was found to have a survival benefit in a murine GBM model (30 days compare to 19 days in control, p<0.01, and 10% of mice being long-term survivors). However, this benefit was only seen when treatment was delivered within the tumor through FcγR-mediated destruction of Tregs. Systemic delivery did not deplete intratumoral Tregs and did not extend survival significantly. Anti-GITR, non-depleting antibodies combined with stereotactic radiation also increases overall survival in murine GL-261 Luc glioma model (67). In GBM patients, selective depletion of Tregs with anti-IL-2Rα mAb during lymphopenia, enhanced response to an EGFRVIII peptide vaccine and improve anti-tumor humoral immunity (68). The effects of anti-Treg therapy in combination with infusion of cytotoxic T cells has not been tested.

## Optimizing Expansion, Engraftment, and Function of T Cells

Current treatment strategies for GBM all have effects on the host immune system. Although many of these effects are immunosuppressive, some of the immune-related changes can be leveraged for improved efficacy of immunotherapy. Experimental data of cancer models and results from metastatic cancer patients suggest addition of radiotherapy to immunotherapy contributes to systemic anti-tumor immunity (69). In murine models, GL261 tumor bearing mice had a median survival of 53 days when treated with PD-1 blockade combined with stereotactic radiosurgery compared to 25-28 days in control animals or those treated with monotherapy (70). In a pre-clinical study evaluating a second generation NKG2D targeting CAR T cell, investigators found that the addition of a single dose of 4 Gy radiation to the tumor resulted in significantly more intra-tumor T cell migration (71). This was associated with increased long-term survival in the SMA-560 glioma model (42% versus 14% with CAR alone, p<0.001). In patients with newly diagnosed high grade glioma, radiation was given 9 days after intra-tumoral administration of adenoviral vector (ADV-TK) as preclinical studies suggest increased efficacy with the combination (72). Twelve patients were treated and 4/4 tumors were found to have CD3 T cell infiltrates on H&E analysis. This was a phase IB study and further investigation is ongoing.

Chemotherapy has also been described to improve engraftment after T cell infusions. Using a murine melanoma tumor model treated with OT-1 T cell infusion followed by OVA peptide vaccination, myeloablation using temozolomide led to a 70-fold expansion of antigen specific T cells compared to controls (28). In the same model, temozolomide-induced lymphopenia increased antigen specific T cell expansion in a dose dependent fashion (73). Interestingly, the immune effects of temozolomide vary based on the dosing schedule. When combined with PD-1 blockade, standard dosing of temozolomide abrogated the survival benefit of PD-1 blockade in murine GL-261 glioma models (74). When the same total dose was delivered in smaller individual doses over a longer period of time (metronomic schedule), the survival benefit of PD-1 blockade was preserved due to avoidance of T cell dysfunction. In a phase II study of DCs loaded with GBM lysate combined with adjuvant temozolomide, CD8 T cells expanded, but the effector memory (CCR7 low, CD45RO high) decreased after the first adjuvant temozolomide dose (75).

The ideal conditioning chemotherapy regimen prior to T cell infusion is controversial. Myeloablative dosing has been shown to increase T cell engraftment. However, myeloablation requires stem cell rescue and is more toxic. A myeloablative dose of temozolomide was tested in B16 F10-OVA melanoma model, in combination with T cell transfer and OVA peptide vaccine improved survival by 10 days compare to non-myeloablative dose (28). The improved survival was mediated by elevated levels of IL-2 post chemotherapy. Higher levels of IL-2 contributed to a significant expansion of transferred T cells and differentiation of naïve T cells to effector T cells with a higher capacity for pro inflammatory cytokine secretion.

Lymphodepletion without myeloablation may be enough. Lymphodepletion prior to CAR T cells targeting EGFRvIII was found to cause regression of tumors and resulted in 50% long term survivors (over 200 days) (29). Animals that received higher temozolomide doses (dose intensified) had enhanced proliferation and persistence of CAR T cells compared to animals receiving the standard dose. Based on this study, phase I clinical trial had been designed to evaluate the anti-tumor efficacy of EGFRvIII CAR T cells after host precondition with dose intensified temozolomide for newly diagnosed GBM patients (NCT02664363). In another phase I clinical trial for patients with recurrent central PNETs, the efficacy of T cell transfer targeting total tumor RNA combined with DC vaccine was evaluated post non myeloablative doses of cyclophosphamide and fludarabine (19). Massive clonal expansion of T cells were found using TCR sequencing. However, the function of these expanded T cells has not been described.

T cell dysfunction is a major limitation of any T cell-based therapy. One strategy to avoid T cell dysfunction is replacement of exhausted and senescent T cells with effector and memory T cells. This replacement can be performed by promoting apoptosis by targeting FOXO4/p53 peptide in senescent T cells (76) and substituting the exhausted T cells with effector and memory T cells using stem cell transplantation (77). Alternatively, dysfunctional T cells can be replaced by T cells recruited by hematopoietic stem cell (HSC) infusion. In a murine GBM model, HSCs were shown migrate to the tumor microenvironment (78). Secretion of chemoattractants such as growth factors and cytokines from tumor cells attract HSCs where HSCs can recruit tumor-specific T cells. A study by Flores et al. demonstrated that HSC infusion after myeloablative RT resulted in homing of tumor specific lymphocytes to the tumor (KR158) *via* CCL3 secretion and tumor control with improved survival (doubling of median survival compared to control) (79).

An alternative is use of induced pluripotent stem cells (iPS) which differentiate into functional T cells (80). For example, murine embryonic fibroblasts were used to create iPS cells using Flt-3 and IL-7 (81). These iPS cells were used to create T cells which were able to reconstitute a normal pool of T cells in a T cell deficient mouse model. Differentiation of T cells from iPS cells can be used as a strategy to produce “rejuvenated” T cells with high proliferative capacity and elongated telomeres (82). Moreover, iPS grown T cells can be transduced with CARs or engineered TCRs specific for tumor antigens (83).

Restoration strategy is another novel area of research. This technique requires harvesting of a functional thymus from cadaveric donor, isolation of thymus organoids and bioengineering them with growth promoting factors and thymo-stimulatory cytokines such as IL-21 (84). These thymic organoids can be transduced with recipient HLA molecules followed by recellularization of bioengineered organoid scaffold to be prepared for transplanting into the recipients (85, 86). These strategies are still experimental and require further study to determine their role in the treatment of patients with GBM.

Preventing or reversing T cell dysfunction will be the key to the future of immunotherapy in the treatment of GBM. Importantly, the effects of standard treatment modalities on the host and exogenously derived T cells will be critical. Manipulation of the peripheral and intra-tumoral immune microenvironment, optimizing the timing and duration of T cell antigen exposure, and providing sufficient T cell activation have the potential to improve responses to immunotherapy. Furthermore, adoptive T cell therapy with antigen specific T cells including CAR T cells or hematopoietic stem cells, are promising approaches to replace dysfunctional T cells in patients with GBM.

## Conclusion

Patients with GBM present with several mechanisms of immunosuppression and T cell dysfunction. Targeted efforts to improve T cell function will result in greater efficacy of platforms such as adoptive T cell transfer and CAR T cell infusion. These efforts include designing T cells that target the major tumor antigens, improving persistence and effector function of T cells, and optimization of tumor microenvironment for the efficacious T cell response in the immunosuppressive tumor setting. The efficacy of immunotherapy for GBM rests on the ability to overcome T cell dysfunction.

## Author Contributions

AK and MR researched data and wrote the article. FD, JH, ES, SN, CY, LJ, and DM contributed to scientific discussion and critical review of the manuscript. SN generated the figures. All authors contributed to the article and approved the submitted version.

## Conflict of Interest

The authors declare that the research was conducted in the absence of any commercial or financial relationships that could be construed as a potential conflict of interest.

## Publisher’s Note

All claims expressed in this article are solely those of the authors and do not necessarily represent those of their affiliated organizations, or those of the publisher, the editors and the reviewers. Any product that may be evaluated in this article, or claim that may be made by its manufacturer, is not guaranteed or endorsed by the publisher.
